# Stroke Mimics: A Case of John Cunningham Virus-Induced Progressive Multifocal Leukoencephalopathy

**DOI:** 10.7759/cureus.70423

**Published:** 2024-09-29

**Authors:** Hafiz Sohail Kamran, Asghar Khan, Clare Siew Boon Ling, Shahid Kausar

**Affiliations:** 1 Medical Enhanced Care Unit, The Dudley Group NHS Foundation Trust (DGFT) Russells Hall Hospital, Dudley, GBR; 2 Acute Medicine, The Dudley Group NHS Foundation Trust (DGFT) Russells Hall Hospital, Dudley, GBR; 3 Medical Enhanced Care Unit, The Dudley Group NHS Foundation Trust, Dudley, GBR; 4 Stroke, Russells Hall Hospital, Dudley, GBR

**Keywords:** cidofovir, cll treatment, hiv aids, jcv encephalitis, mefloquine, progressive multifocal leukoencephalopathy (pml)

## Abstract

Progressive multifocal leukoencephalopathy (PML) is a rare, often fatal neurological disorder caused by the John Cunningham virus (JCV). It affects immunocompromised individuals, leading to brain demyelination. Diagnosis involves MRI scans and JCV detection in cerebrospinal (CSF) fluid. The mortality rate is high, and current intervention focusses on reversing immunosuppression. We report a patient with chronic lymphocytic leukaemia (CLL) who was diagnosed with PML. He is a 66-year-old male with CLL presenting with multiple falls, right arm weakness, and cognitive impairment. Following MRI head scans and CSF analysis, he was diagnosed with PML. Treatment for CLL was deemed inappropriate due to immunosuppression risk. We initiated Levetiracetam to prevent seizures and considered mirtazapine to prevent viral spread. Mefloquine and cidofovir were considered, but the patient chose not to commence on them. He was discharged with multidisciplinary support. In conclusion, we advise that these stroke-like symptoms may necessitate comprehensive investigation beyond initial CT scans, as exemplified by this case of PML. Relying solely on radiological findings may overlook rare conditions, and clinical skills such as a good history and examination should still be prioritised.

## Introduction

Progressive multifocal leukoencephalopathy (PML) is a rare but fatal neurological disorder that is caused by the polyomavirus known as John Cunningham virus (JCV). It is estimated that JCV is present in the large majority of the general population without causing any symptoms. However, reactivation of the virus leading to disease primarily affects immunocompromised individuals, such as those with HIV, lymphoproliferative disease, or patients undergoing treatment with immunosuppressive or immunomodulatory therapies. This contributes to clinical features, including cognitive dysfunction, hemianopsia, aphasia, ataxia, seizures, and motor or sensory dysfunction. Although brain biopsy remains the gold standard modality to detect JCV, other resources like CT scans, MRIs, and blood/serological tests can help in reaching the diagnosis/assessment of further progression. MRI typically shows T2-hyperintense and T1-hypointense lesions in the subcortical white matter of the brain or cerebellar peduncles. Patients having JCV infection can present to the stroke team in the hospital with stroke-like symptoms, i.e., weakness in certain parts of the body, slurring of speech, etc. That is why it is very important to look into other causes as well that can present as stroke-like symptoms, especially if patients have a background of certain conditions, i.e., CLL/HIV infection/transplant patients, etc., that make them prone to having certain conditions like JCV infection. In addition to clinical suspicion, initial investigations such as MRIs and brain scans can prompt medical professionals to investigate alternative causes, particularly if MRI changes do not align with an acute stroke. Here, we report a patient with chronic lymphocytic leukaemia (CLL) who was diagnosed with PML and presented to the stroke ward.

## Case presentation

Patient description

A 66-year-old male patient with known CLL presented to his regular haematology follow-up appointment with a two-week history of multiple falls, right arm weakness, and cognitive impairment. The patient was diagnosed with small lymphocytic lymphoma in 2019 and has been under regular monitoring by the haematology team without needing treatment.

Investigation results

**Figure 1:**
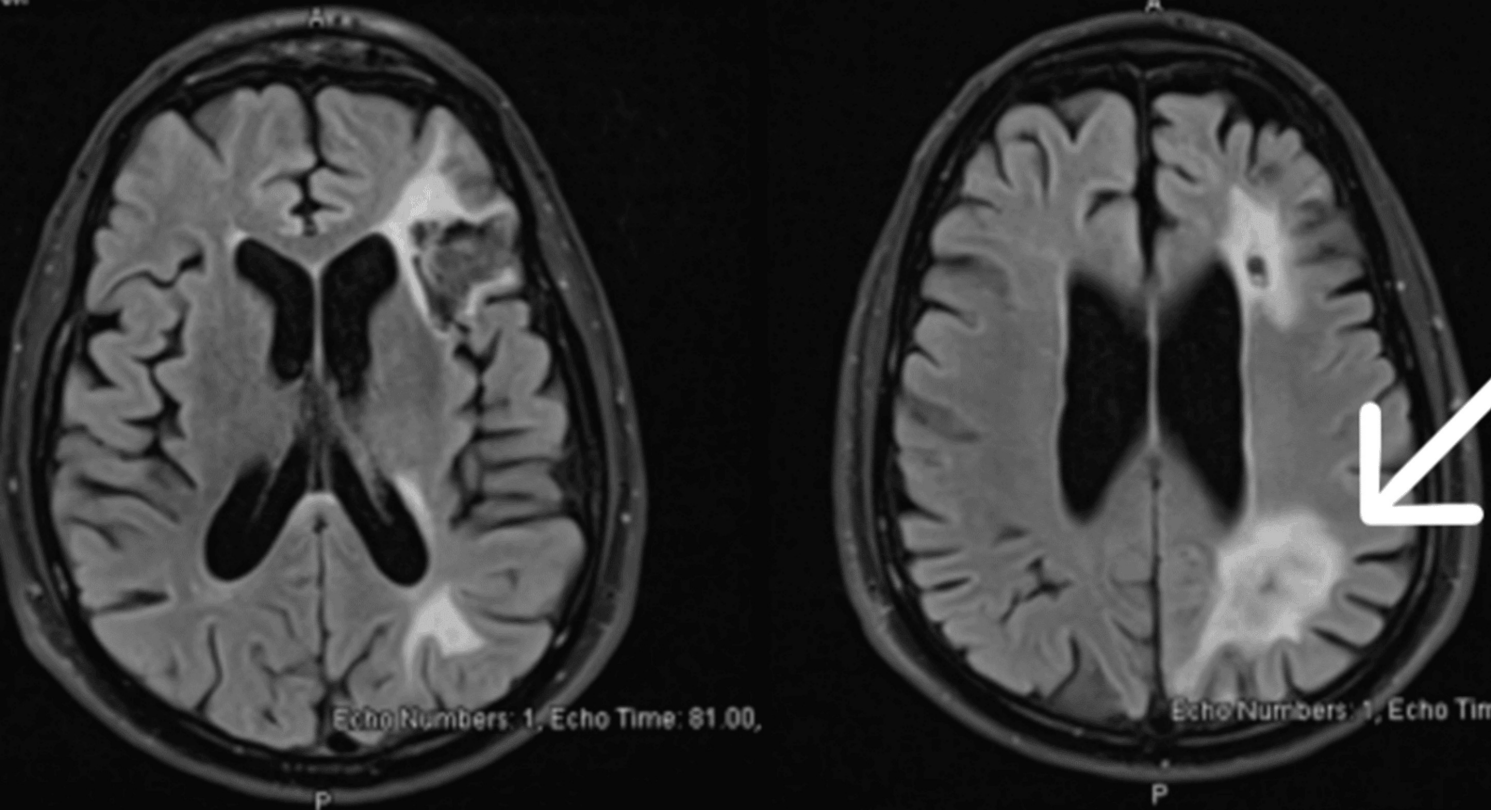
MRI brain on admission

**Figure 2:**
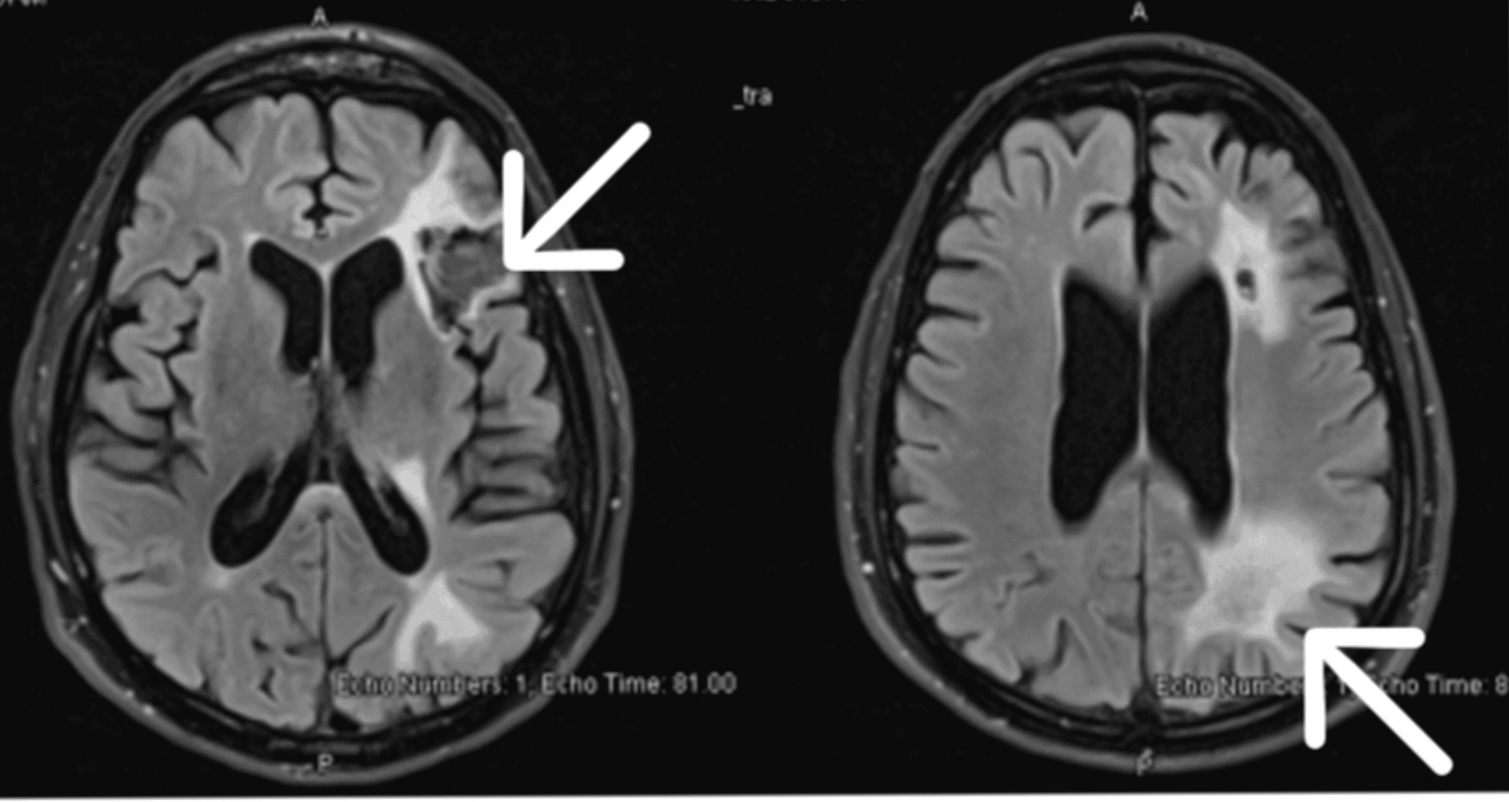
MRI brain three weeks later

**Figure 3:**
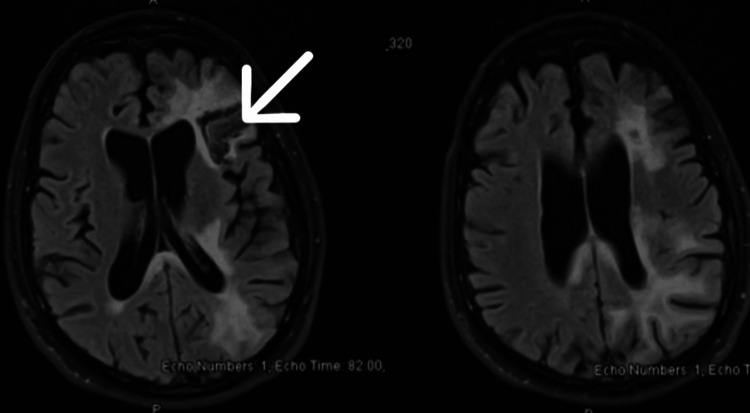
MRI brain at two months

**Figure 4:**
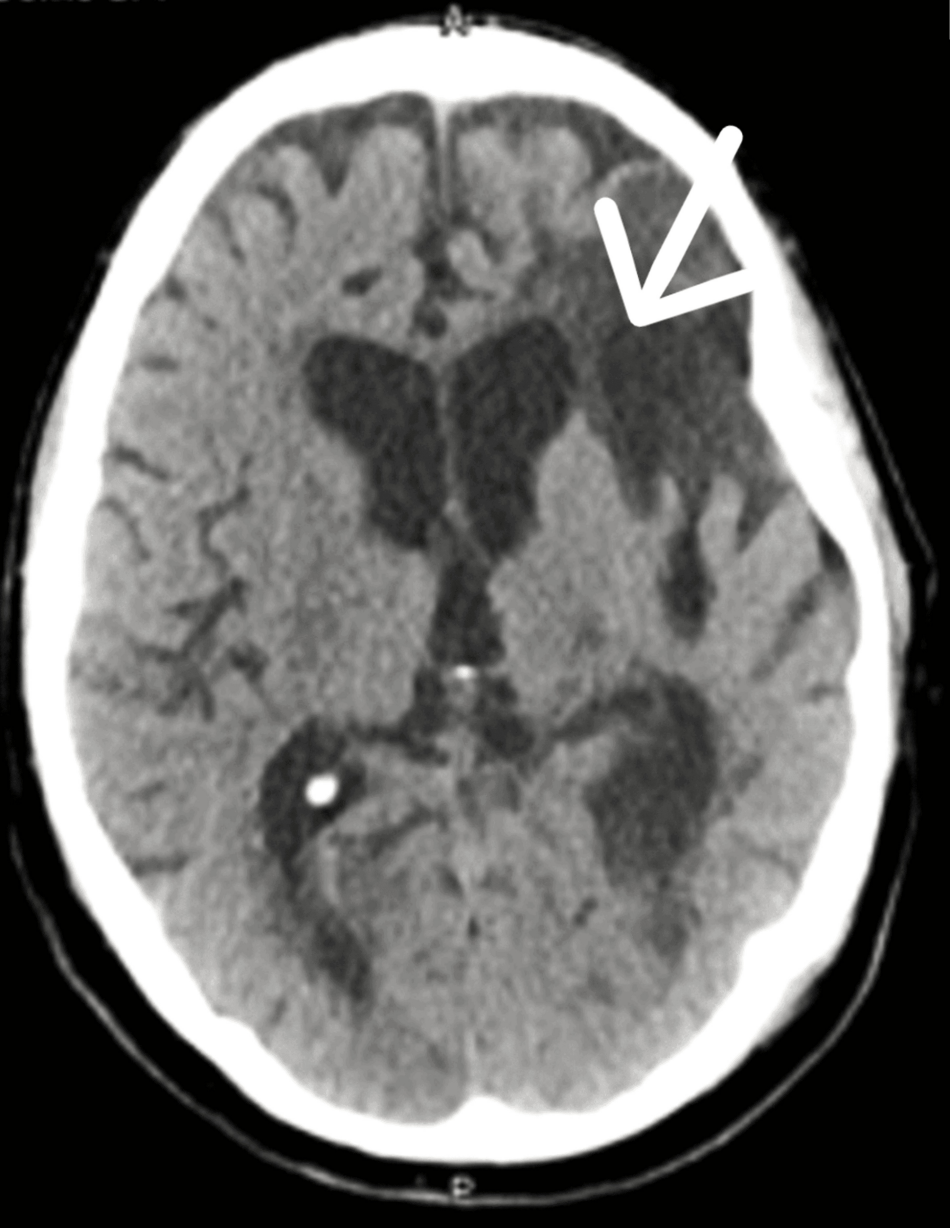
CT scan brain after one year

On examination, the patient had reduced power with hyperreflexia in his right upper limb and hyperreflexia with upgoing plantar reflex in the left lower limb. He scored 7/10 on the abbreviated mental test (AMT), showing probable cognitive impairment. Vital observations were unremarkable. The patient was admitted to the acute medical team and later referred to the stroke team for further investigations of a likely stroke. An initial CT head scan revealed low density in the left frontal and parietal parenchyma, which was in keeping with previous cerebral infarcts. Further evaluation was required with an MRI head scan (Figure 1). MRI head with contrast (Figure 2) showed abnormal T2/FLAIR hyperintensity in the subcortical and deep white matter of the left parietal lobe, extending into the posterior left frontal lobe. There was a further abnormality in the splenium of the corpus callosum on the right, which demonstrated some degree of mass effect. In order to look for the progression of the changes in the brain parenchyma, another MRI brain was arranged at a two-month interval (Figure 3). A series of blood investigations (biochemical, autoimmune panel, and microbiological) were performed to look into the causes that are mentioned in Table 1. This was reviewed by the haematology team, who suggested a lumbar puncture and cytocentrifuge (Cytospin). Lumbar puncture was positive for JC virus (Papovavirus group antibodies - JCV DNA detected; Table 2). Following a discussion between the neurology and haematology teams, it was felt that PML was not an indication for the treatment of CLL. Treatment of CLL would cause further immunosuppression; hence, it would not be a rational decision. The patient was started on levetiracetam daily for seizure prevention. We suggested starting him on mirtazapine to slow down the progression of the viral spread. Mefloquine and cidofovir were also considered, albeit with poor evidence basis, for these treatments. Due to concerns about the potential side effects of mefloquine and cidofovir, the patient decided against these medications. The patient has been on only mirtazapine daily. He was eventually discharged to the community with multidisciplinary input. The patient remained under the joint care of neurology and haematology. A continued neurological and functional decline was observed in patient status over time, supported by a progressive change in brain parenchyma, as evident in the CT scan of the brain done one year later (Figure 4).

**Table 1:** Investigations

Blood test results (on admission)		Normal range
Na	137	133–146 mmol/L
K	4.6	3.5–5.3 mmol/L
Creatinine	111	50–98 umol/L
eGFR	60	>90
CRP	<1	0–5 mg/L
Hb	126 g/L	115–165g/L
WBC	10.3 x 10^9^/L	4–11 x 10^9/L
Platelets	106 x 10^9^/L	150–450 x 10^9/L
Neutrophils	4.48 x 10^9^/L	2–7.5 x 10^9/L
Lymphocyte	4.53 x 10^9^/L	1.5–4.5 x 10^9/L
Monocytes	1.10 x 10^9^/L	1.5–4.5 x 10^9/L
ESR	42	1–10 mm/hr
Other blood test results		
ACE	43 U/L	20–70 U/L
Kappa	30.50 mg/L	3.30–19.40 mg/L
Lambda	23.90 mg/L	5.71–26.30 mg/L
Kappa/Lambda ratio	1.276	0.260–1.650
Lupus anticoagulant screen ratio	2.58	0.8–1.2
Lupus overall test ratio	2.6	0.8–1.2
Lupus anticoagulant	Strong positive	
IgG cardiolipin abs	1 GPL-U/ml	<10 GPL-U/ml
Proteinase 3 Abs	<0.2 IU/ml	<0.2 IU/ml
Myeloperoxidase Abs	<0.2 IU/ml	<0.2 IU/ml
dsDNA	0.8 IU/ml	<10 IU/ml
Antinuclear Abs	Positive 1:320 homogenous	
IgG	15.40 g/L	6–16 g/L
IgA	1.90 g/L	0.8–4 g/L
IgM	2.31 g/L	0.5–2 g/L
Paraprotein	4.7g/L	
HIV	Negative	

**Table 2:** Cerebrospinal result

Lumbar puncture results	
CSF glucose	3.6 mmol/L
CSF protein	0.33 g/L
CSF cytospin	No cells, no evidence of CLL
Papovavirus group antibodies	JC virus DNA detected
Treponemal antibodies (syphilis screen)	Negative

## Discussion

JCV is estimated to be present in 70-90% of the general population [1]. However, it can manifest clinically only in certain individuals, i.e., immunocompromised. JCV targets the myelin-producing cells (oligodendrocytes) of the central nervous system, causing progressive demyelination of the white matter [2]. Although stereotactic brain biopsy remains the gold standard for diagnosis, practically, diagnosis is predominantly made via a combination of magnetic resonance imaging scans and detection of JCV in cerebrospinal fluid. The mortality rate remains high at 30-50% in the first few months following diagnosis [3]. Currently, the main intervention involves a reversal of immunosuppression to restore the normal host response to the virus [4].

This case highlights several issues regarding the diagnosis of stroke mimics and the presentation of PML. First, this case emphasises how crucial it is to maintain a vigilant and open-minded approach when diagnosing patients with stroke-like symptoms. It is imperative to remain receptive to various imaging and investigative methods to distinguish between stroke and other potential conditions. For example, the atypical MRI findings were the key to prompting us to explore other diagnostic possibilities. Second, we have learned that PML can also affect seemingly immunocompetent individuals. Although there are certain drugs like Mefloquine and Cidofovir that can be tried as treatment options, their specific effects on the disease were difficult to recognise in previous studies. PML primarily affects individuals with compromised immune systems. Most notably those with congenital or acquired immunodeficiency conditions (e.g., AIDS), patients with haematological or solid malignancies, or individuals undergoing immune-modulating therapies [5,6]. Our patient had a history of haematological malignancy but did not receive chemotherapy or immunotherapy. While CLL can result in secondary immunosuppression due to impaired humoral and cellular immunity, it does not significantly elevate the risk of PML on its own [7]. Instances of PML occurring in seemingly immunocompetent individuals are exceedingly rare. The precise mechanism behind JCV reactivation in non-immunodeficient patients remains a subject of controversy. In a case study [6] in the United States, a healthy patient diagnosed with PML experienced a full recovery after a six-month mefloquine trial. Likewise, a case was documented [7] in Norway featuring an immunocompetent patient who received intravenous cidofovir following a PML diagnosis. He exhibited clinical improvement, and a reduction of white matter lesions was observed on MRI scans. However, the author stated that spontaneous recovery cannot be ruled out and that the progression of lesions could be due to disease progression instead. Therefore, additional research and clinical trials must be conducted to optimise the prognostic outcome for patients with PML.

## Conclusions

This case demonstrates that a thorough investigation is often required in patients presenting with stroke-like clinical features. Often, in clinical practice, patients are dismissed following a CT head that does not correlate with ischaemic disease. This indicates that radiological findings are not sufficient in diagnosing a rare disease such as PML. Instead, a good history, examination, and investigations, such as an LP, are crucial in rare diagnoses. Also, this case study highlights the importance of further research into treatment for PML, as it is still predominantly a disease with a very poor prognosis.

## References

[1] Shackelton LA, Rambaut A, Pybus OG, Holmes EC (2006). JC virus evolution and its association with human populations. J Virol.

[2] Saribas AS, Ozdemir A, Lam C, Safak M (2010). JC virus-induced progressive multifocal leukoencephalopathy. Future Virol.

[3] (2024). Progressive multifocal leukoencephalopathy [Internet]. https://www.ninds.nih.gov/health-information/disorders/progressive-multifocal-leukoencephalopathy.

[4] (2024). Progressive multifocal leukoencephalopathy/JC virus infection. https://clinicalinfo.hiv.gov/en/guidelines/hiv-clinical-guidelines-adult-and-adolescent-opportunistic-infections/progressive.

[5] Moenster RP, Jett RA (2012). Mirtazapine and mefloquine therapy for progressive multifocal leukoencephalopathy in a patient infected with human immunodeficiency virus. Am J Health Syst Pharm.

[6] Tan IL, Koralnik IJ, Rumbaugh JA, Burger PC, King-Rennie A, McArthur JC (2011). Progressive multifocal leukoencephalopathy in a patient without immunodeficiency. Neurology.

[7] Naess H, Glad S, Storstein A, Rinaldo CH, Mørk SJ, Myhr KM, Hirsch H (2010). Progressive multifocal leucoencephalopathy in an immunocompetent patient with favourable outcome. A case report. BMC Neurol.

